# ER Ca^2+^ overload activates the IRE1α signaling and promotes cell survival

**DOI:** 10.1186/s13578-023-01062-y

**Published:** 2023-07-03

**Authors:** Song Zhao, Haiping Feng, Dongfang Jiang, Keyan Yang, Si-Tong Wang, Yu-Xin Zhang, Yun Wang, Hongmei Liu, Caixia Guo, Tie-Shan Tang

**Affiliations:** 1 State Key Laboratory of Membrane Biology, Institute of Zoology, Chinese Academy of Sciences, Beijing, 100101 China; 2 Beijing Institute of Genomics, Chinese Academy of Sciences/China National Center for Bioinformation, Beijing, 100101 China; 3 Beijing Institute for Stem Cell and Regenerative Medicine, Beijing, 100101 China; 4 University of Chinese Academy of Sciences, Beijing, 100049 China

**Keywords:** ER Ca^2+^ overload, TMCO1, ER stress, IRE1α

## Abstract

**Background:**

Maintaining homeostasis of Ca^2+^ stores in the endoplasmic reticulum (ER) is crucial for proper Ca^2+^ signaling and key cellular functions. Although Ca^2+^ depletion has been known to cause ER stress which in turn activates the unfolded protein response (UPR), how UPR sensors/transducers respond to excess Ca^2+^ when ER stores are overloaded remain largely unclear.

**Results:**

Here, we report for the first time that overloading of ER Ca^2+^ can directly sensitize the IRE1α-XBP1 axis. The overloaded ER Ca^2+^ in TMCO1-deficient cells can cause BiP dissociation from IRE1α, promote the dimerization and stability of the IRE1α protein, and boost IRE1α activation. Intriguingly, attenuation of the over-activated IRE1α-XBP1s signaling by a IRE1α inhibitor can cause a significant cell death in TMCO1-deficient cells.

**Conclusions:**

Our data establish a causal link between excess Ca^2+^ in ER stores and the selective activation of IRE1α-XBP1 axis, underscoring an unexpected role of overload of ER Ca^2+^ in IRE1α activation and in preventing cell death.

**Supplementary Information:**

The online version contains supplementary material available at 10.1186/s13578-023-01062-y.

## Introduction

As a major intracellular Ca^2+^ store, the ER is essential for protein folding, secretion and Ca^2+^ homeostasis [1]. Environmental stressors, such as disruption of Ca^2+^ homeostasis, viral infection, and redox homoeostasis, lead to the accumulation of misfolded proteins in the ER, eliciting ER stress [2, 3]. To adapt to the disturbance of the external environment and reestablish ER homeostasis, the unfolded protein response (UPR) is triggered through three adaptive pathways meditated by the ER stress sensors, including inositol-requiring enzyme 1 (IRE1α/ERN1), activating transcription factor 6 (ATF6), and protein kinase RNA-like-ER kinase (PERK/EIF2AK3) [2, 4–8]. Under physiological conditions, the ER luminal domain of three stress effectors is associated with binding immunoglobulin protein (BiP) to maintain their inactive state [5, 9].

IRE1α is the most conserved and extensively studied UPR sensor protein [10, 11], which belongs to the type I transmembrane protein family, consisting of a luminal domain (LD), a single transmembrane domain, and a dual enzyme domain including a kinase domain (Kinase) and an endoribonuclease (RNase) domain in the cytosolic portion [3, 12]. Once activated, BiP is titrated away by unfolded proteins, leaving IRE1α free to initiate dimerization-dependent autophosphorylation of its cytosolic domain. The subsequent allosteric activation of the cytosolic RNase domain triggers unconventional splicing of the mRNA encoding the transcription factor X-box binding protein 1 (XBP1), thereby promoting translation of the functional transcription factor, XBP1s (spliced XBP1). In turn, XBP1s then upregulates the transcription of conserved genes encoding ER-associated degradation (ERAD) components, chaperones, and folding enzymes to re-establish the ER proteostasis [5, 13–15]. Under severe or chronic ER stress, prolonged IRE1α activation promotes apoptotic signaling downstream of the ASK-JNK axis and inflammatory signaling downstream of nuclear factor (NFkB) by binding tumor necrosis factor receptor-associated factor 2 (TRAF2) to promote cell death [16, 17].

It is known that depletion of ER Ca^2+^ with the sarco-/endoplasmic-reticulum Ca^2+^-ATPase (SERCA) inhibitor thapsigargin (TG) can lead to ER dysfunction and to activate the UPR [2, 18]. To maintain efficient folding, the ER maintains an environment enriched in chaperones [19]. Previous studies have identified the Ca^2+^ binding potential of many chaperones, such as BiP, GRP94, and calreticulin, which act as buffer proteins for maintaining the ER Ca^2+^ homeostasis, while Ca^2+^ in turn is also important for the activity of these chaperones [20, 21]. ER Ca^2+^ depletion could significantly affect protein processing and then lead to the accumulation of unfolded proteins in the ER [22–24]. Most of the accepted models propose that BiP binds directly to the regulator lumen domain of the UPR sensors to keep them in an inhibited state [25, 26], while ER Ca^2+^ depletion-triggered accumulation of unfolded proteins can compete to bind BiP and thus to activate the UPR pathway [5, 25].

ER Ca^2+^ overload happens in multiple diseases including Alzheimer’s disease [27–32], targeting of intracellular Ca^2+^ stores may have therapeutic potential for treating these diseases. However, whether and how UPR sensors/transducers respond to the excess Ca^2+^ when ER stores are overloaded remains unclear. As an ER Ca^2+^ load-activated Ca^2+^ channel (CLAC), transmembrane and coiled-coil domains 1 (TMCO1) is important for maintaining ER Ca^2+^ homeostasis [33], whose deletion can cause ER Ca^2+^ overload. We and others have reported that TMCO1 deficiency can cause cerebrofaciothoracic dysplasia (CFTD) [34], defects in bone formation [35] and defects in corpus callosum development [36]. In this study, we explored the effect of TMCO1 deficiency on ER stress signaling. We show that depletion of TMCO1 can activate IRE1α by increasing its stability and oligomerization in a Ca^2+^-dependent manner. Moreover, the ER Ca^2+^ overfilling signaling directly sensitizes the IRE1α-XBP1 axis, which is essential for modulating cell fate and helping cell survival.

## Results

### TMCO1 depletion boosts the activation of the IRE1α-XBP1 pathway

**Fig. 1:**
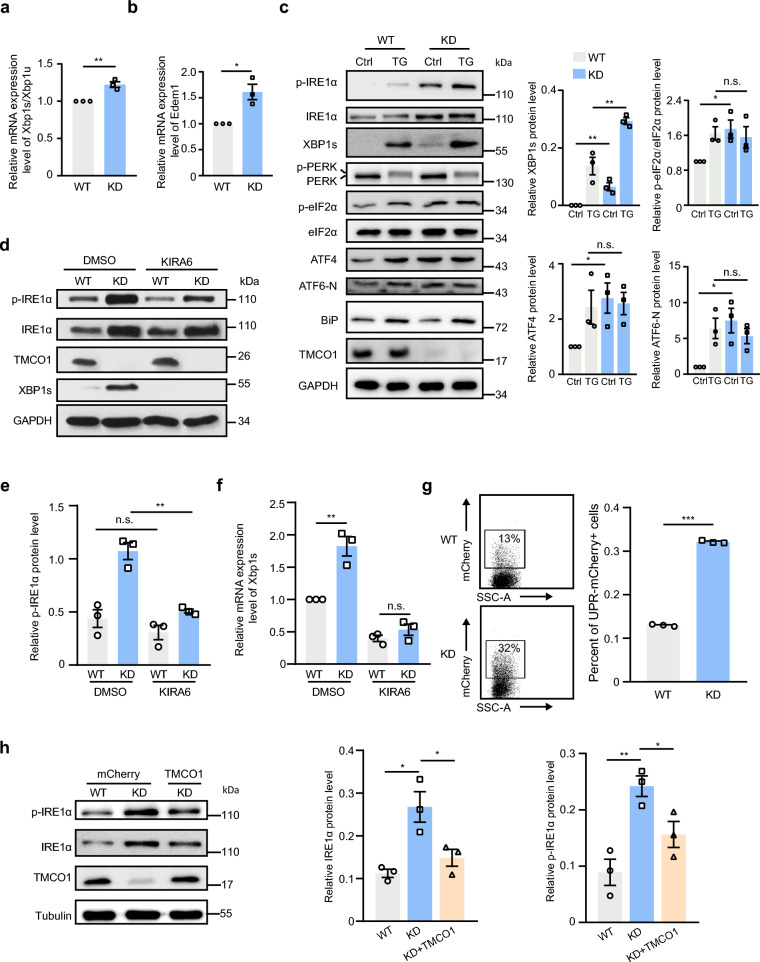
TMCO1 knockdown leads to the overactivation of IRE1α. **a**–**b** qRT-PCR analysis of Xbp1s/Xbp1u (**a**), and Edem1 (**b**) mRNA levels in WT or TMCO1 KD cells. Data are shown as mean ± SEM from three independent experiments. n.s., no significance, *P < 0.05, ** P < 0.01. **c** Western blotting analysis of the UPR marker proteins, including p-PERK, PERK, p-eIF2α, eIF2α, ATF4, p-IRE1α, IRE1α, XBP1s and ATF6-N (N-terminal cleavage product of ATF6), in untreated or TG-treated (1 μM, 4 h) WT and TMCO1 KD cells. GAPDH was used as a loading control. Relative quantification of each protein was shown in the right panel. Bar graphs represent the mean ± SEM from three independent experiments. *P < 0.05, **P < 0.01, n.s., no significance. **d** Western blotting analysis of proteins in WT and TMCO1 KD cells treated with/without 20 μM KIRA6 for 24 h. GAPDH was used as a loading control. **e** Relative quantification of p-IRE1α protein levels in D. Data are shown as the mean protein intensity normalized to GAPDH ± SEM from 3 independent experiments. n.s., no significance, **P < 0.01. **f** qRT-PCR analysis of Xbp1 mRNA splicing in WT and TMCO1 KD cells treated with/without 20 μM KIRA6 for 24 h. Bar graphs represent the mean ± SEM from three independent assays. **P < 0.01, n.s., no significance. **g** FACS analysis of mCherry-positive cells. WT or TMCO1 KD cells were transfected with the UPRE-mCherry. SSC-A: side-scatter area, which can indicate the granularity of the cell. Bar graph in the right panel represents the mean ± SEM for mCherry-positive cells from three independent assays. ***P < 0.001. **h** Western blotting analysis of p-IRE1α and IRE1α levels in WT and TMCO1 KD cells transfected with control mCherry vector or TMCO1-IRES-mCherry. Tubulin was used as a loading control. Relative quantification of protein levels is shown in the right panel. Data are shown as the mean protein intensity normalized to tubulin ± SEM from 3 independent experiments. *P < 0.05, **P < 0.01

To assess whether TMCO1 deficiency is linked to ER stress, we investigated the role of TMCO1 in the induction of the UPR. Interestingly, we found that knockdown (KD) of TMCO1 could increase the expression of IRE1α and promote IRE1α overactivation and XBP1 splicing (Additional file 1a and Fig. 1a), however, with no obvious effect on the expression of BiP and CHOP (Additional file 1a, b, c), two key factors involved in the ATF6 and PERK pathways of the UPR, respectively [15, 37]. In support of this, TMCO1 KD also significantly elevated the mRNA level of *Edem1*, a classical XBP1s target gene (Fig. 1b). To analyze the effects of TMCO1 deficiency on the UPR pathways more accurately, WT and KD cells were exposed to ER stressor TG. Supportingly, when cells were challenged by TG, TMCO1 KD boosted the activity of IRE1α but not PERK and ATF6 (Fig. 1c). Given that KD of TMCO1 promotes IRE1α expression and the RNase activity of IRE1α, which can selectively degrade some mRNAs through a process known as regulated IRE1-dependent decay (RIDD) [38], we then examined the mRNA level of a RIDD target gene SPARC [39, 40]. We found that its expression was significantly downregulated in KD cells (Additional file 1d). These results indicate that TMCO1 depletion can lead to an active ER stress pathway mediated mainly by IRE1α but not PERK or ATF6.

To further explore the association of TMCO1 loss with IRE1α-mediated UPR, we treated TMCO1 wild-type (WT) and KD HeLa cells with an IRE1α inhibitor KIRA6 [41]. We found that KIRA6 treatment markedly reduced the levels of IRE1α phosphorylation and XBP1s in KD cells with no obvious effect on IRE1α expression (Fig. 1d, e, f), indicating that TMCO1 KD directly activates both the kinase and RNase activities of IRE1α. To detect cellular ER stress-induced IRE1α activation directly, we then constructed a UPR-mCherry reporter with the UPR response element (UPRE), which can drive mCherry expression through XBP1s production, thus reflecting IRE1α activation specifically [42, 43].The reporter was transfected into HEK-293 T cells followed by treatment with either TG (to induce ER stress) or 4μ8C (a specific IRE1α RNase inhibitor) [44]. The quantity of TG used for the cell treatment was calibrated first to avoid excessive XBP1 splicing, and a concentration of 1 μM TG for a 2 h incubation was defined for ER stress stimulation, as no significant difference of spliced XBP1s levels was observed between WT and KD under this condition (Additional file 1e). We found that TG treatment significantly stimulated the expression of XBP1s and mCherry compared to the DMSO control, while supplementation with 4μ8C largely blocked the stimulatory effect, indicating that this UPR-mCherry reporter we prepared could be used to indicate the ER stress-induced IRE1α activation in cells (Additional file 1f) as previously reported. We then transfected the UPR-mCherry reporter into WT and KD cells and detected a significant increase in the mCherry signal in KD cells compared to WT cells (Fig. 1g), supporting that TMCO1 loss can boost ER stress-induced IRE1α activation. ER Ca^2+^ overload and activation of IRE1α induced by knockdown of TMCO1 were also verified in HEK-293 T cells (Additional file 1 g, h, i).

To further support that the overactivation of IRE1α in TMCO1 KD cells is indeed caused by TMCO1 depletion, a TMCO1-IRES-mCherry construct was transfected back into the TMCO1 KD cells. We found that expressing TMCO1-IRES-mCherry but not mCherry in KD cells could attenuate the increase in IRE1α expression and its phosphorylation (Fig. 1h). Taken together, these results indicate that TMCO1 plays an important regulatory role in IRE1α-XBP1s pathway activation.

### TMCO1 regulates the activation of IRE1α depending on Ca^2+^ signaling

**Fig. 2:**
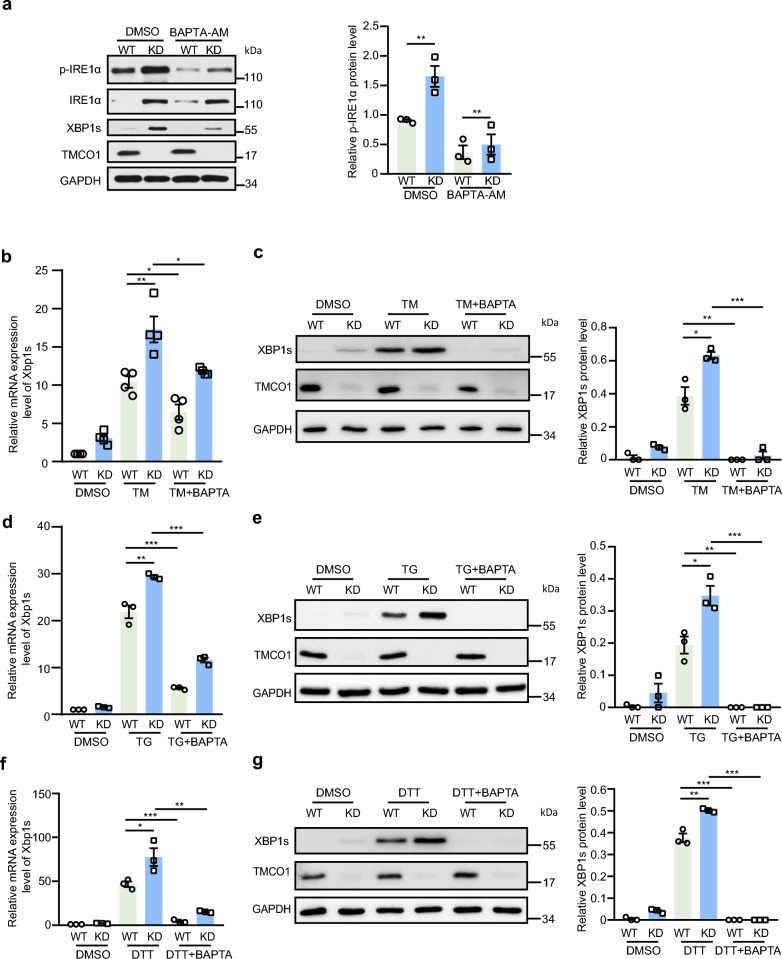
TMCO1 KD enhances IRE1α-XBP1 pathway in a Ca.^2+^ dependent manner. **a** Western blotting analysis of p-IRE1α, IRE1α and XBP1s levels in WT and TMCO1 KD cells treated with/without 50 μM BAPTA-AM for 2 h. Relative quantification of p-IRE1α level is shown in the right panel. Data are shown as the mean protein intensity normalized to GAPDH ± SEM from 3 independent experiments. **P < 0.01. **b** qRT-PCR analysis of Xbp1 mRNA splicing in WT and TMCO1 KD cells treated with 3 μg/ml TM along with/without 50 μM BAPTA-AM for 4 h. Bar graphs represent the mean ± SEM from three independent assays. *P < 0.05; **P < 0.01. **c** The levels of XBP1s in WT and TMCO1 KD cells were measured by western blotting analysis after the treatment of 3 μg/ml TM along with/without 50 μM BAPTA-AM for 4 h. GAPDH was used as a loading control. Relative quantification of XBP1s levels was shown in the right panel. Data are shown as the mean protein intensity normalized to GAPDH ± SEM from 3 independent experiments. *P < 0.05, **P < 0.01, ***P < 0.001. **d** qRT-PCR analysis of Xbp1 mRNA splicing in WT and TMCO1 KD cells treated with 1 μM TG along with/without 50 μM BAPTA-AM for 4 h. Bar graphs represent the mean ± SEM from three independent assays. **P < 0.01, ***P < 0.001. **e** The levels of XBP1s in WT and TMCO1 KD cells were measured (Left) and quantified (Right) by western blotting after the treatment of 1 μM TG along with/without 50 μM BAPTA-AM for 4 h. GAPDH was used as a loading control. Data are shown as the mean protein intensity normalized to GAPDH ± SEM from 3 independent experiments. *P < 0.05, **P < 0.01, ***P < 0.001. **f** qRT-PCR analysis of Xbp1 mRNA splicing in WT and TMCO1 KD cells treated with 5 mM DTT along with/without 50 μM BAPTA-AM for 4 h. Bar graphs represent the mean ± SEM from three independent assays. *P < 0.05; **P < 0.01, ***P < 0.001. **g** The levels of XBP1s in WT and TMCO1 KD HeLa cells were measured (Left) and quantified (Right) by western blotting analysis after the treatment of 5 mM DTT along with/without 50 μM BAPTA-AM for 4 h. GAPDH was used as a loading control. Data are shown as the mean protein intensity normalized to GAPDH ± SEM from 3 independent experiments. **P < 0.01, ***P < 0.001

We then investigated how TMCO1 regulates the IRE1α-XBP1s activation. Considering TMCO1 depletion can cause an ER Ca^2+^ overload, we wonder whether the abnormal Ca^2+^ signaling plays a role in IRE1α overactivation in KD cells. Using BAPTA-AM, an intracellular Ca^2+^ chelator, to treat WT and KD cells, we noted that the overactivation of IRE1α phosphorylation and increased expression of XBP1s protein in KD cells were largely abrogated (Fig. 2a), indicating that the overactivation of IRE1α caused by TMCO1 deficiency is likely attributed to altered Ca^2+^ levels.

To determine whether TMCO1 depletion could sensitize the IRE1α-XBP1 pathway upon ER stress induction, and whether alteration of Ca^2+^ level is involved in this process, WT and KD cells were exposed to three distinct ER stressors independently, including tunicamycin (TM), TG or DTT, in the presence or absence of BAPTA-AM. We found that treatments with these ER stressors could significantly increase the mRNA (Fig. 2b, d, f) and protein (Fig. 2c, e, g) levels of XBP1s in both WT and KD cells, with a more pronounced increase in KD cells compared to WT cells. Interestingly, concurrent treatment with BAPTA-AM almost completely abolished the increase of XBP1s caused by ER stressors. These data demonstrate that TMCO1 deficiency can enhance the IRE1α-XBP1 axis in a Ca^2+^ signaling-dependent manner. Additionally, we observed that the effect of BAPTA-AM on XBP1s protein level was far greater than that on its mRNA level, which could be due to the direct effect of BAPTA-AM on protein synthesis by upregulating phosphorylation of the translational initiation factor eIF2α [45], resulting in a suppression of the initiation step of protein synthesis. We also demonstrated that BAPTA-AM could repress global translation (Additional file 2a). Thus, it is likely that BAPTA-AM inhibits the translation of XBP1s.

### ER Ca^2+^ overfilling sensitizes the IRE1α-XBP1 axis

Although TM, DTT and TG treatment could sensitize the IRE1α-XBP1 pathway in a Ca^2+^ signaling-dependent manner in KD cells, it is unclear whether this sensitization effect was caused by the overfilling of ER Ca^2+^ or the release of excessive Ca^2+^ into the cytoplasm from ER. Our findings that TG, an ER stressor that can deplete ER luminal Ca^2+^ [46], but not TM and DTT, a reducing agent that prevents disulfide bond formation of ER nascent proteins [47], caused a transient release of Ca^2+^ from the ER ( Additional file 2b, c), provide preliminary evidence of the involvement of ER Ca^2+^ in this process. We therefore speculated that the overfilling of Ca^2+^ in the ER caused by TMCO1 depletion might affect the IRE1α activation. To support this notion, different Ca^2+^ inducers other than TG, including ATP, ionomycin (iono) and carbachol (Cch), were used to treat WT and KD cells, and then the cytosolic Ca^2+^ was measured by the Ca^2+^ indicator fura-2. Significantly higher amplitudes of Ca^2+^ transients were detected in KD cells than in WT cells upon treatment with these Ca^2+^ inducers (Additional file 3a, b, c). Moreover, the overfilling state of the ER Ca^2+^ store was further confirmed by the mKate-linker-G-CEPIA1er (miGer) [48] in KD cells (Additional file 3d). Together, these results confirm that TMCO1 deficiency indeed causes overfilling of ER Ca^2+^ store.

**Fig. 3:**
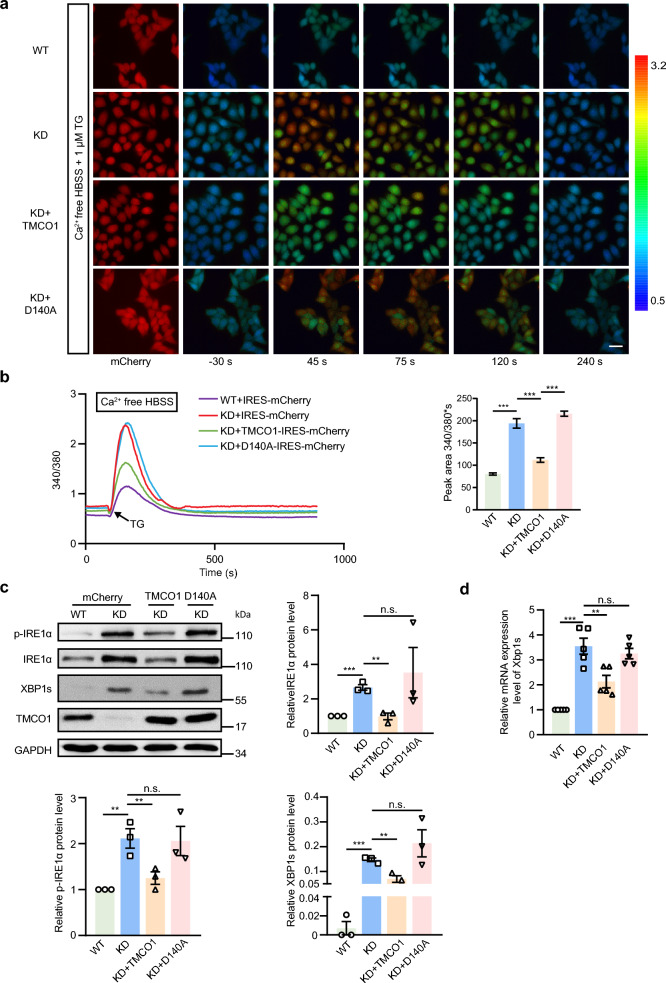
Over-activation of IRE1α induced by TMCO1 knockdown is dependent on Ca^2+^ rather than the TMCO1 protein. **a** Representative images of Fura-2 340/380 ratios in WT, TMCO1 KD cells and TMCO1 KD cells transfected with TMCO1 or TMCO1-D140A-IRES-mCherry. Ratios were recorded for 1 μM TG-induced Ca^2+^ transients in these cells. The pseudo-color calibration scale for 340/380 ratios was shown on the right. mCherry images (first column) were captured before TG-induced Ca^2+^ imaging to identify TMCO1 expressing cells. Representative images were shown for cells 30 s before (second column), and 45 s, 75 s, 120 s, and 240 s after application of 1 μM TG as indicated. Scale bar, 20 μm. **b** Left panel, 1 μM TG-triggered Ca^2+^ transients in WT transfected with IRES-mCherry (purple trace line, n = 124), TMCO1 KD transfected with IRES-mCherry (red trace line, n = 76), TMCO1 KD cells transfected with TMCO1-IRES-mCherry (green trace line, n = 62), TMCO1 KD cells transfected with TMCO1-D140A-IRES-mCherry (blue trace line, n = 173). Each trace line in B is an average of Ca^2+^ responses from all mCherry-positive cells in each group. Right panel, statistical analysis of the average peak area of 1 μM TG-triggered Ca^2+^ mobilization curves. Bar graphs represent the mean ± SEM from three independent assays. ***P < 0.001. **c** Western blotting analysis of p-IRE1α, IRE1α and XBP1s levels in WT, TMCO1 KD cells, TMCO1 KD cells transfected with TMCO1 or TMCO1-D140A-IRES-mCherry. GAPDH was used as a loading control. Relative quantification of each protein levels was shown in the right panel. Bar graphs represent the mean ± SEM from three independent experiments. **P < 0.01, ***P < 0.001, n.s., no significance. **d** qRT-PCR analysis of Xbp1 mRNA splicing in WT, TMCO1 KD HeLa cells, TMCO1 KD HeLa cells transfected with TMCO1 or TMCO1-D140A-IRES-mCherry. Bar graphs represent the mean ± SEM from three independent assays. **P < 0.01, ***P < 0.001, n.s., no significance

We then explored the role of ER Ca^2+^ overfilling in the activation of IRE1α. Based on our previous results [33], a TMCO1 mutant (D140A) lacking the Ca^2+^ permeation function was constructed. We found that the increased Ca^2+^ release after TG treatment in KD cells could be rescued by the expression of WT TMCO1 but not the D140A mutant, demonstrating that the regulation of Ca^2+^ signaling by TMCO1 depends on its Ca^2+^ permeability (Fig. 3a, b). Interestingly, we found that exogenous expression of WT TMCO1 but not the D140A mutant could rescue the phosphorylation level of IRE1α (Fig. 3c) and the expression of XBP1s in KD cells (Fig. 3c, d). These data clearly show that the overload of ER Ca^2+^ is critical for the regulatory role of TMCO1 in IRE1α activation, and the activation of IRE1α is closely linked to the Ca^2+^ overfilling state in the ER instead of the TMCO1 protein itself.

### Overload of ER Ca^2+^ disrupts the interaction between BiP and IRE1α

**Fig. 4:**
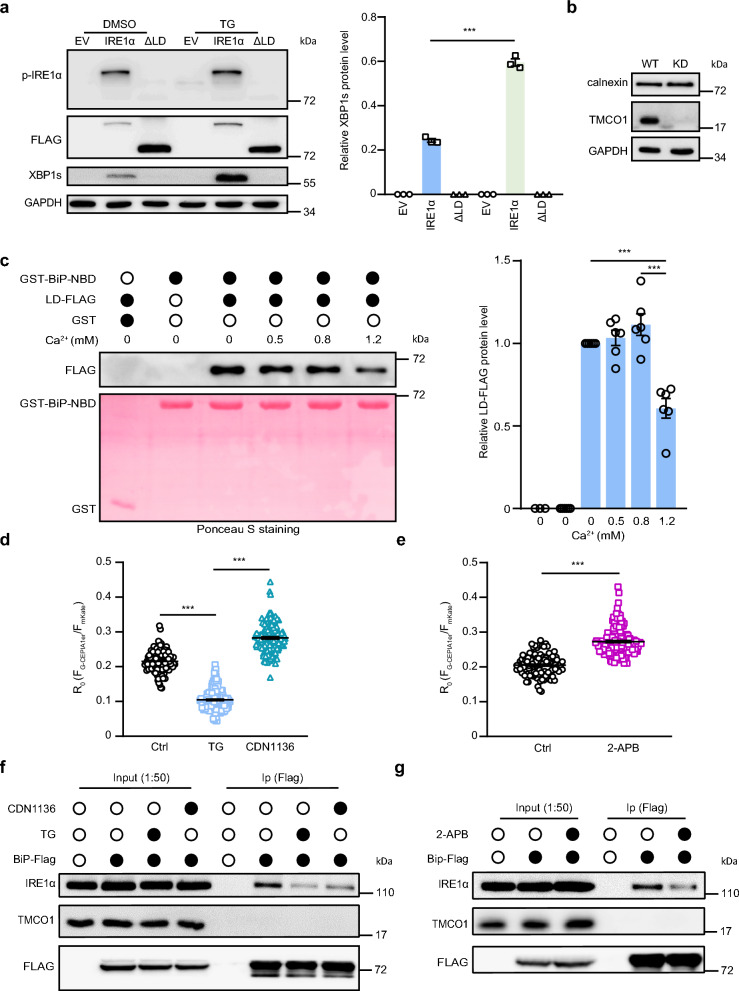
ER Ca^2+^ overload reduces the interaction between IRE1α and BiP. **a** The levels of XBP1s in HEK293T-IRE1α knockout cells transfected with IRE1α or IRE1α deleted LD were measured after treating with 1 μM TG for 2 h, followed by western blotting analysis. GAPDH was used as a loading control. Relative quantification of XBP1s levels was shown in the right panel. Data are shown as the mean protein intensity normalized to GAPDH ± SEM from 3 independent experiments. ***P < 0.001. EV, empty vector. ∆LD, deleted LD of IRE1α. **b** Expression levels of calnexin were analyzed by western blotting in the wild-type or TMCO1 KD cells. GAPDH was used as a loading control. **c** Pull down assay assessing the effects upon addition of increasing concentrations of Ca^2+^ to GST-tagged BiP-NBD and FLAG-tagged IRE1α-LD complex. 1.2 mM Ca^2+^ disrupted IRE1α-luminal domain interaction and causes the BiP-ATPase domain to dissociate. Relative quantification of LD-FLAG level was shown in the right panel. Data are shown as mean ± SEM from three independent experiments. Statistical analysis was performed using one-way ANOVA with Tukey’s multiple comparison test: ***P < 0.001. **d** The resting G-CEPIA1er fluorescence ratio signals were detected in HEK293T treated with 1 μM TG or 5 μM CDN1136 for 2 h. Ctrl, n = 103. TG, n = 171. CDN1136, n = 160. Bar graphs represent the mean ± SEM from three independent assays. ***P < 0.001. **e** The resting G-CEPIA1er fluorescence ratio signals were detected in HEK-293 T treated with/without 100 μM 2-APB for 2 h. Ctrl, n = 105. 2-APB, n = 165. Bar graphs represent the mean ± SEM from three independent assays. ***P < 0.001. (**f **and **g**) HEK-293 T cells transfected with BiP-Flag were treated with 1 μM TG or 5 μM CDN1136 (**f**) or 100 μM 2-APB (**g)** for 2 h followed by a co-immunoprecipitation for Flag. Protein loading was normalized to FLAG and relative co-immunoprecipitated IRE1α was examined via immunoblotting

We next investigated how the overload of ER Ca^2+^ affects the IRE1α activation. BiP is a negative regulator of IRE1α, whose dissociation from the luminal domain (LD) of IRE1α is required for IRE1α activation [49, 50]. Consistent with previous results [51], deletion of the LD (resides 30-407) in IRE1α attenuated IRE1α phosphorylation and XBP1 splicing (Fig. 4a), supporting that the LD of IRE1α was important for IRE1α activation. Normally, the activation of the UPR pathway induced by the accumulation of unfolded proteins in ER leads to the elevation of chaperones in response to the excessive accumulation of unfolded proteins in ER. However, the expression of both BiP (Additional file 1a) and calnexin, another ER resident chaperone with a high capacity for Ca^2+^ binding [52, 53] remained unchanged when TMCO1 was knocked down (Fig. 4b), indicating that no accumulation of large amounts of unfolded protein in the ER occur upon TMCO1 depletion.

We then examined whether ER Ca^2+^ overload could affect the interaction between BiP and IRE1α. Previous studies have reported that the association between BiP and IRE1α is mediated by the nucleotide binding domain in BiP and the LD of IRE1α in a nucleotides-independent manner [54, 55]. We therefore purified both the GST-tagged BiP-nucleotide binding domain (NBD) and Flag-tagged IRE1α-LD separately followed by GST pull-down experiment in the presence of different concentrations of Ca^2+^. As the estimated Ca^2+^ concentration in the ER lumen is around 0.5 to 0.8 mM in the resting state [26, 56], three different concentrations of Ca^2+^, that is, 0.5, 0.8 and 1.2 mM, were used in the pull-down assay. Our result showed that the interaction between BiP-NBD and IRE1α-LD could only be disrupted by 1.2 mM Ca^2+^ but not by 0.8/0.5 mM Ca^2+^ (Fig. 4c), indicating that the binding of NBD to IRE1α’s luminal domain is dependent on Ca^2+^ and that only Ca^2+^ beyond the physiological range could disrupt the interaction between BiP and IRE1α.

To support that, we also examined the effect of a simulated ER Ca^2+^ overload environment on the interaction between BiP and IRE1α. To that end, HEK-293 T cells were treated with CDN1163, a pharmacologic agent known to increase ER Ca^2+^ levels by inducing SERCA pump activation [53], or 2-APB, an antagonist that blocks the exit of ER Ca^2+^ by antagonizing IP_3_Rs [57], with TG as a negative control. As expected, TG reduced ER Ca^2+^ levels, whereas CDN1136 and 2-APB increased ER Ca^2+^ levels (Fig. 4d, e). Our immunoprecipitation results showed that BiP interacted with IRE1a rather than TMCO1 in the resting state, and TG or CDN1136/2-APB treatment significantly reduced the association of BiP to IRE1α (Fig. 4f, g), indicating that either depletion or overload of Ca^2+^ in the ER can reduce the interaction of BiP with IRE1α. Taken together, these results reveal that ER Ca^2+^ overload caused by depletion of TMCO1 can reduce the binding of BiP to IRE1α and boost the activation of IRE1α.

### ER Ca^2+^ overfilling enhances the stability and oligomerization of IRE1α

**Fig. 5:**
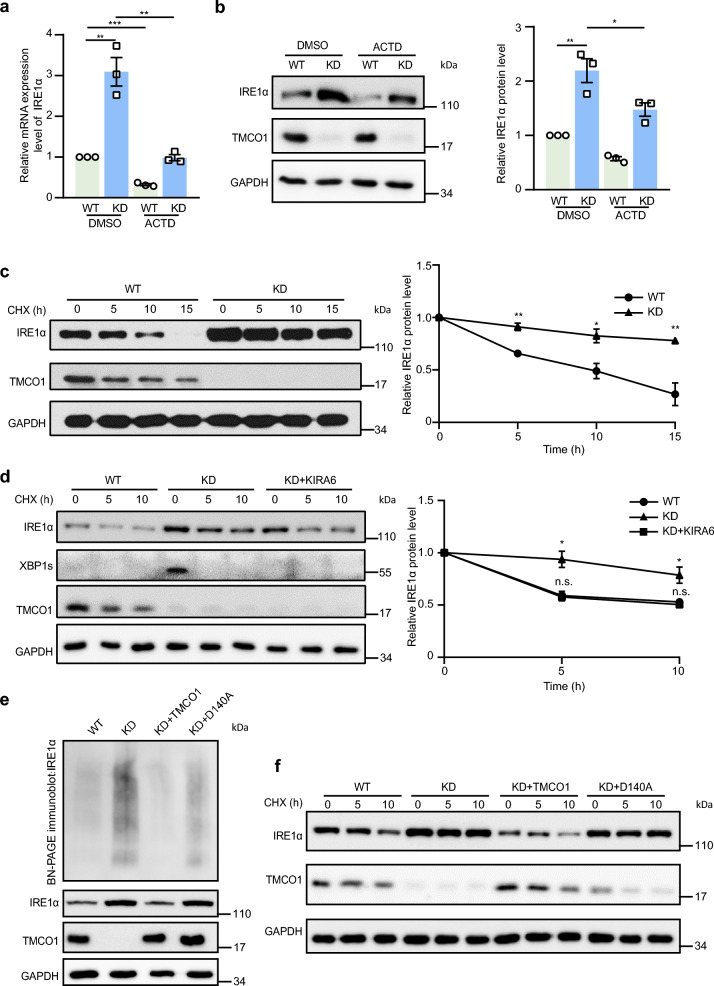
The dimerization and stability of IRE1α regulated by TMCO1 depends on the overload of ER Ca^2+^ store. **a** qRT-PCR analysis of IRE1α mRNA in WT or TMCO1 KD cells treated with/without 2.5 μM ACTD for 5 h. Bar graphs represent the mean ± SEM from three independent assays. **P < 0.01, ***P < 0.001. **b** Western blotting analysis of IRE1α levels in WT and TMCO1 KD cells treated with/without 2.5 μM ACTD for 5 h. GAPDH was used as a loading control. Relative quantification of IRE1α levels was shown in the right panel. Bar graphs represent the mean ± SEM from three independent experiments. *P < 0.05; **P < 0.01. **c** The stability of IRE1α was measured after the treatment of 100 μg/ml CHX for the indicated times, followed by western blotting analysis. GAPDH was used as a loading control. Relative quantification of IRE1α levels was shown in the right panel. Bar graphs represent the mean ± SEM from three independent experiments. *P < 0.05, **P < 0.01. **d** The stability of IRE1α was measured after the treatment of 100 μg/ml CHX along with/without 20 μM KIRA6 for the indicated times, followed by western blotting analysis. GAPDH was used as a loading control. Relative quantification of IRE1α levels was shown in the right panel. Bar graphs represent the mean ± SEM from three independent experiments. *P < 0.05, n.s., no significance. **e** The digitonin lysate of WT, TMCO1 KD cells, TMCO1 KD cells transfected with TMCO1 or TMCO1-D140A-IRES-mCherry were analyzed by BN-PAGE immunoblotting with IRE1α antibody. GAPDH was used as a loading control. **f** The stability of IRE1α in WT, TMCO1 KD cells. TMCO1 KD cells transfected with TMCO1 or TMCO1-D140A-IRES-mCherry were treated with 100 μg/ml CHX for the indicated times, followed by immunoblotting. GAPDH was used as a loading control

Since KD of TMCO1 could increase the expression of IRE1α (Additional file 1a), we then explored the underlying mechanism. To determine whether the increased IRE1α expression was due to its enhanced transcription, KD cells were treated with actinomycin D (ACTD), an inhibitor of RNA synthesis [58, 59]. qPCR and western blotting results showed that ACTD treatment significantly decreased the transcription of IRE1α (Fig. 5a), while the increased IRE1α expression at protein levels could still be detected in TMCO1 KD cells (Fig. 5a, b), suggesting that TMCO1 depletion may promote IRE1α protein stability at the post-transcriptional level. To determine whether TMCO1 depletion can promote IRE1α expression at the protein level, we treated WT and KD cells with cycloheximide (CHX), a widely used protein synthesis inhibitor in eukaryotic cells [60]. We found that the half-life of IRE1α protein was dramatically increased in TMCO1 KD cells (Fig. 5c), demonstrating that depletion of TMCO1 can markedly enhance the stability of IRE1α protein. It has been reported that IRE1α protein can undergo dimer/oligomerization and is stabilized under ER stress [61, 62]. To examine whether the oligomerization of IRE1α was related to the increased stability of IRE1α caused by TMCO1 depletion, cells were treated with CHX in the presence or absence of KIRA6, which could prevent IRE1α oligomerization. We found that the half-life of IRE1α was dramatically decreased in KD cells after treatment with KIRA6 (Fig. 5d), providing evidence for the involvement of IRE1α oligomerization in its enhanced stability. We next analyzed IRE1α oligomerization in TMCO1-deficient cells by Blue Native polyacrylamide gel electrophoresis (BN-PAGE) immunoblotting [62]. The results showed that knockdown of TMCO1 significantly increased IRE1α oligomerization, which could be completely reverted by WT TMCO1 but not the D140A mutant (Fig. 5e), revealing a Ca^2+^-dependent enhancement of IRE1α oligomerization in KD cells. Furthermore, consistent with the IRE1α oligomerization results, the increased half-life of IRE1α protein in KD cells was dramatically decreased by WT TMCO1 but not by the D140A mutant (Fig. 5f). Together, our results support that the IRE1α stability and its dimerization/oligomerization are regulated by overload of ER Ca^2+^ when TMCO1 get depleted.

### *Tmco1* deficiency enhances IRE1α signaling under ER stress in vivo

**Fig. 6:**
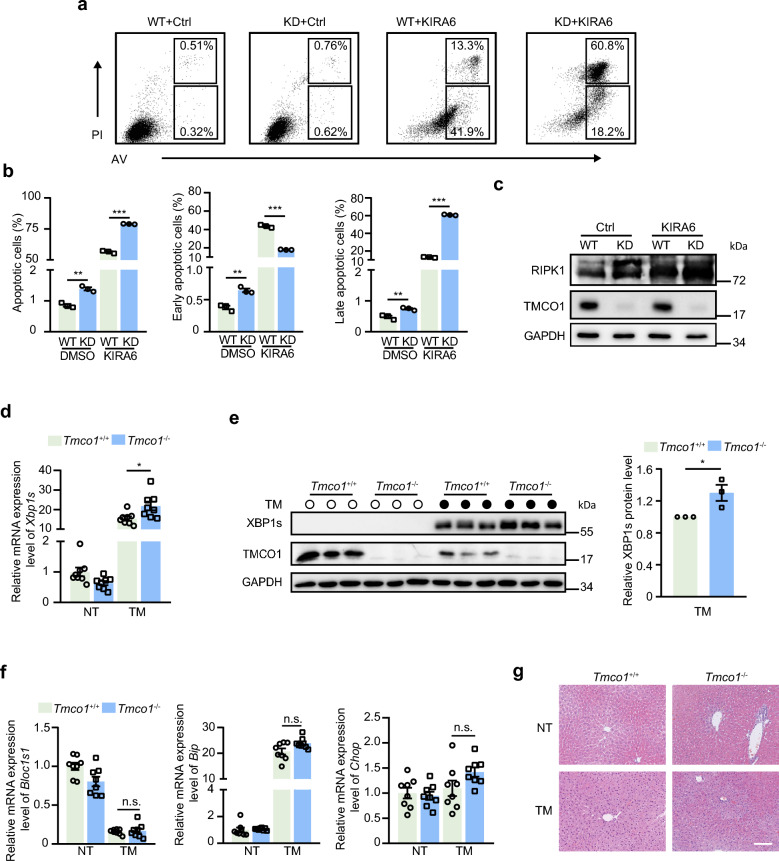
TMCO1 knockdown sensitizes IRE1α-XBP1 axis to promote cell survival. **a** Representative annexin V-FITC and PI staining in WT or TMCO1 KD cells treated with/without 20 μM KIRA6 for 24 h followed by flow cytometry. AV, annexin V. **b** The proportion of dead cells was counted in A. Bar graphs represent the mean ± SEM from three independent assays. **P < 0.01, ***P < 0.001. **c** Western blotting analysis of proteins in WT and TMCO1 KD cells treated with/without 20 μM KIRA6 for 24 h. GAPDH was used as a loading control. **d** qRT-PCR analysis of *Xbp1s* in the liver from *Tmco1*^+/+^ *and Tmco1*^−/−^mice at 2 months of age injected intraperitoneally with TM (2 μg/g) for 8 h. Each point represents independent animal. Untreated mice, NT: n = 8; TM treated mice: n = 8. *P < 0.05, n.s., no significance. **e** The levels of XBP1s in the livers from *Tmco1*^+/+^ and *Tmco1*^−/−^ mice injected intraperitoneally with TM (2 μg/g) for 8 h were measured through immunoblotting. GAPDH was used as a loading control. Quantification of the relative expression of XBP1s was shown in the right panel. Bar graphs represent the mean ± SEM from three independent experiments. Each point represents independent animal. *P < 0.05. **f** qRT-PCR analysis of *Bloc1s1*, *Bip*, *Chop* in the liver from *Tmco1*^+/+^ *and Tmco1*^−/−^mice injected intraperitoneally with TM (2 μg/g) for 8 h. Each point represents independent animal. Untreated mice, NT: n = 8; TM treated mice: n = 8. n.s., no significance. **g** Representative images of H&E staining of liver tissues from *Tmco1*^+/+^ *and Tmco1*^−/−^ mice injected with Tm for 8 h (magnification × 200). Scale bar, 100 μm. Three animals per group were analyzed

IRE1α activation has been reported to enhance the susceptibility of cells to apoptosis. In view of the pro-survival properties of XBP1 splicing [1], we set out to test whether IRE1α activation induced by TMCO1 deficiency could promote cell survival. WT and KD cells were treated with KIRA6 for 24 h and then the early apoptotic, late apoptotic, and dead cells were quantified by flow cytometry using Annexin V/PI. The results showed that although TMCO1 knockdown could promote cell death to a certain level, inhibiting the IRE1α pathway by KIRA6 significantly boosted cell death, with a significant more effect in KD cells than WT cells (Fig. 6a, b). Specifically, KD cells induced a significantly higher percentage of both early and late apoptotic (necrotic stage) cells compared with WT cells under basal conditions, whereas upon KIRA6 treatment, KD lead to significantly more late apoptotic cells than WT. Consistent with annexin V-FITC + PI staining results, the level of RIPK1, an important mediator of necrosis [63] was also significantly elevated in KD cells in the presence of KIRA6 compared to the control cells (Fig. 6c). These results indicate that the potentiation of IRE1α-XBP1 axis could help cell survival when ER Ca^2+^ get overloaded in KD cells.

To test the effect of TMCO1 on IRE1α signaling in vivo, *Tmco1*^+/+^ and *Tmco1*^−/−^ mice at 2 months of age were intraperitoneally injected with a single dose of TM to trigger a strong UPR reaction in livers [1, 64–68]. Mice were sacrificed at 8, 16 or 24 h after TM injection for comparison of IRE1α signaling activation in the liver. Consistent with previous studies [66], the highest amounts of XBP1s were observed at 8 h at both mRNA (Additional file 4a) and protein (Additional file 4b) levels, suggesting a decay of the TM-induced XBP1 splicing occurs in vivo. Therefore, the following analysis of IRE1α signaling in the liver was performed at 8 h after TM injection. Our results showed that both the mRNA and protein levels of XBP1s were increased after TM treatment, with a bigger increase in *Tmco1*^−/−^ mice compared to WT mice (Fig. 6d, e). However, when analysis of the mRNA levels of genes involved in other UPR downstream signaling pathways, including *Bloc1s1*, *Bip* and *Chop*, was performed in WT and *Tmco1*-deficient livers, no significant change was detected under basal and TM treated situations (Fig. 6f). These results support that TMCO1 deficiency specifically enhances the IRE1α-XBP1 pathway under ER stress conditions in vivo. Furthermore, we also assessed the damage effect of the TM injection in the liver tissue. Both hematoxylin and eosin (H&E) staining as well as picrosirius red staining showed that there were no signs of hepatocyte damage in *Tmco1*^+/+^ and *Tmco1*^−/−^ mice, indicating that the dose of TM used to treat mice did not cause liver damage at the histological level (Fig. 6g and Additional file 4c). Interestingly, although TMCO1 deficiency failed to upregulate basal ER stress in livers from 2-month-old mice, we detected a significant increase of XBP1s in the liver of 8-month-old *Tmco1*^−/−^ mice compared with age-matched WT mice (Additional file 4d). Given that the pro-survival properties of XBP1s could ameliorate Alzheimer's disease by improving synaptic function and proteostasis and protect against obesity-linked metabolic deterioration [37, 69], it is plausible that the activation of XBP1s in 8-month-old *Tmco1*^−/−^ mice may promote cell survival during mouse aging. Taken together, our results show that TMCO1 depletion boost the activation of the IRE1α-XBP1 pathway under ER stress to promote cell survival in vivo*.*

## Discussion

**Fig. 7:**
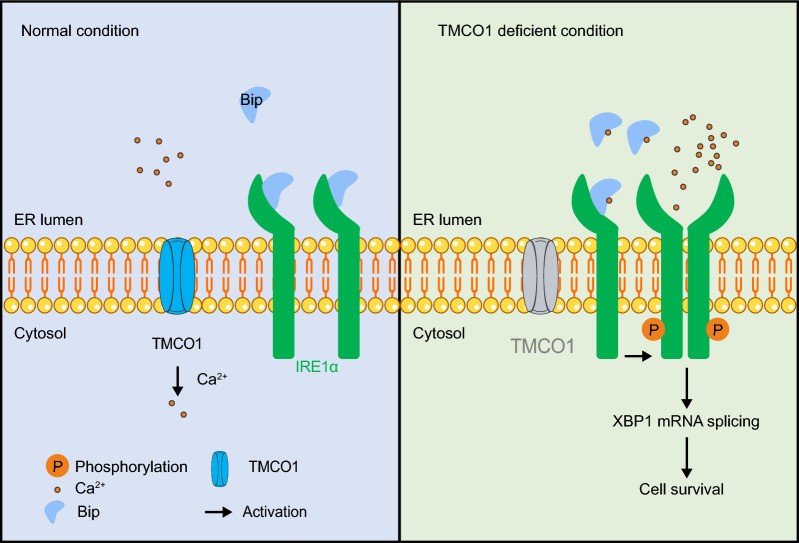
Graphical Abstract for TMCO1 deficiency-induced the activation of IRE1α-XBP1s axis. Graphical Abstract: under basal conditions, the monomeric inactive state of IRE1α is maintained through the interaction with chaperone BiP. In the case of TMCO1 knockdown, the induced overfilling of Ca^2+^ signaling decreases the interaction between IRE1α and BiP, increases IRE1α dimerization and then promotes cell survival through sensitizing IRE1α-XBP1 axis

IRE1α is the most conserved and well-studied UPR sensor, which determines cell fate under ER stress derived from many factors, including inflammation, oxidative stress, glucose deprivation and aberrant Ca^2+^ regulation [70, 71]. IRE1α expression is therefore under stringent regulation in vivo. Here, we identified TMCO1 as a novel regulator of IRE1α. Based on our results that TMCO1 deficiency can cause the overload of ER Ca^2+^, which then disrupts the interaction between BiP and IRE1α, we propose that the reduced interaction after TMCO1 depletion promotes the dimerization and stability of IRE1α so that IRE1α easily reaches a priming state in response to possible external stimuli to promote cell survival (Fig. 7). Our findings highlight a new way of regulating IRE1α by TMCO1, and establish a causal link between excess Ca^2+^ in ER stores and the selective activation of IRE1α-XBP1 axis.

Maintaining homeostasis of Ca^2+^ stores in the ER is crucial for proper Ca^2+^ signaling and key cellular functions such as protein synthesis, folding and maturation. ER Ca^2+^ homeostasis is modulated by Ca^2+^ binding proteins (e.g., calreticulin, BiP, and GRP94), ER-resident Ca^2+^ transporters, and Ca^2+^ channels, including ryanodine receptors (RyRs), ER-resident inositol 1,4,5-trisphosphate receptor (IP_3_Rs), and SERCA [72–75]. It is known that ER Ca^2+^ depletion caused by SERCA inhibitors, such as TG, can trigger a robust activation of ER stress [76, 77]. Of note, alterations in ER Ca^2+^ homeostasis include not only Ca^2+^ depletion but also Ca^2+^ overfilling, with the latter being also an important ER Ca^2+^ homeostatic feature. However, it is still unclear whether ER Ca^2+^ overload induces ER stress. We previously reported that deficiency of TMCO1 in mouse granulosa cells could activate ER stress mainly mediated by the activities of the IRE1α pathway [78], it is still unclear whether the effect is cell type-specific and related to ER Ca^2+^ overload. Here in this study, using in vitro and in vivo mouse model of ER stress, we have demonstrated that the overloading ER Ca^2+^ caused by TMCO1 depletion directly sensitizes the IRE1α-XBP1 axis. Therefore, our findings underscore an unexpected role of overload of ER Ca^2+^ in ER stress regulation.

Despite disagreements over details, most UPR regulatory models acknowledge the importance of BiP-substrate interactions [25]. It is well recognized that BiP binds to the UPR sensors to restrict it in an inhibited state [79, 80]. Under the condition of ER stress, unfolded proteins replace BiP from UPR sensors, leading to their activation [54]. The components of the ER protein folding machinery, including calnexin [26, 81], calreticulin [82], Grp94 [83, 84] and PDI, are known to have low-affinity Ca^2+^-binding sites, whose occupancy is likely decreased upon ER Ca^2+^ depletion within the physiological range [26]. Therefore, ER Ca^2+^ depletion is expected to favor the dissociation of BiP from UPR sensors [26, 85]. However, TMCO1 deficiency did not result in an increased expression of chaperones in the ER (Additional file 1a and Fig. 4b). Distinct from the fashion that ER Ca^2+^ depletion activates UPR sensors through accumulation of unfolded proteins in the ER, activation of UPR sensors by ER Ca^2+^ overload is more related to its function to directly disrupt the BiP-IRE1α interaction (Fig. 4c, f, g).

Given that BiP is highly promiscuous in its client specificity [86], we could not exclude the possibility that high Ca^2+^ also affects the BiP-PERK/ATF6 interactions. BiP has also been shown to bind Ca^2+^ [87, 88], though the designated Ca^2+^-binding sites have not been identified. It seems that Ca^2+^ binding to BiP helps BiP-substrate interaction, and Ca^2+^-depletion in ER destabilizes the BiP-substrate interaction. For example, Ca^2+^ and ATP have been shown to bind to BiP in a cooperative manner which is essential for the peptide binding and folding abilities of this BiP chaperone [26, 89]. ER Ca^2+^ is required for the binding capacity of BiP for ER-resident SREBP2 and prevents its exit from the ER [53]. Changes in BiP’s substrate interaction kinetics are observed within minutes of ER Ca^2+^ depletion [90]. ER Ca^2+^ depletion can cause TCRα protein’s dissociation from BiP thus facilitating its exit from ER [26]. Our present results showed that high ER Ca^2+^ level (ER Ca^2+^ overload) disrupts BiP interaction with IRE1α (Fig. 4c), suggesting that excessive Ca^2+^ binding to BiP also destabilizes BiP-protein interactions. Here, using TMCO1 KD cells, we report a model of UPR regulation in which the three UPR branches are regulated divergently: IRE1α is activated while PERK and ATF6 are attenuated. These data support that TMCO1 knockdown preferentially sensitizes the IRE1α-XBP1 pathway, although the exact mechanism needs further investigation. Therefore, both ER Ca^2+^ overload and Ca^2+^ depletion could cause ER stress response, although they function through distinct mechanisms.

Activation of IRE1α induced by TMCO1 depletion or *Tmco1* knockout appears to be a life-versus-death determination in both cells and mice. IRE1α has long been identified as a positive regulator of cell survival. Conversely, the repression of IRE1α is related to potentiating apoptosis [71, 91, 92]. Autophosphorylation of IREα activates its RNase domain to cleave XBP1 mRNA, producing a potent transcription factor called XBP1s [13]. Target genes of XBP1s encode proteins that promote ER protein folding capacity and quality control [93]. IRE1α improves cell adaptation by enhancing XBP1 mRNA splicing. Moreover, following the formation of high-order clusters, IRE1α can interact with some proteins, including the proapoptotic BCL-2 family members BAX and BAK, the scaffold proteins TRAF2, TRAF6, and RACK1, and STAT3, potentially to regulate apoptosis, inflammation, and proliferation [16, 44, 94–101]. The result that knockdown of TMCO1 resulted in a lower level of XBP1 splicing compared to TG-treated cells, indicates that the activation of IRE1α caused by TMCO1 depletion is in the adaptive UPR stage and is more likely to promote cell survival (Additional file 1e). TMCO1 knockdown could lead to the increase of apoptosis, and the activation of IRE1a induced by ER Ca^2+^ overload is mainly to resist the deterioration from early apoptosis to late apoptosis (Fig. 6a, c).

In summary, this study establishes a model of ER stress caused by ER Ca^2+^ overload and identified TMCO1 as a novel regulator of IRE1α. We define the increased protein stability of IRE1α regulated by ER Ca^2+^ as a new concept of the priming state and provide insights into further understanding of IRE1α activation.

## Conclusions

Our findings not only identify TMCO1 as a novel regulator of IRE1α, but also highlight an unexpected role of overload of ER Ca^2+^ in ER stress regulation, which represents a novel model of UPR signaling activation mediated by excess ER Ca^2+^. Given the central role of IRE1α in the regulation of cell fate and in the pathology of various diseases, establishing a causal link between TMCO1 depletion and ER Ca^2+^ overfilling with IRE1α will help the discovery of drugs targeting the UPR signaling.

## Materials and methods

### Antibodies, chemicals, plasmids, and primers

The sources of antibodies, chemicals, plasmids, and primers used in this study can be found in Additional file 5.

### Animals

The *Tmco1*^+/- ^mouse strain (C57BL/6 J) was generated in Shanghai Model Organisms Center, Inc. (Shanghai, China), as described previously [78]. The male wild type, *Tmco1*^+/+^ (C57BL/6 J) or *Tmco1*^−/−^ mice aged 8 weeks were used for all experiments. Mice were housed in a temperature-controlled environment with a 12-h light/dark cycle and free access to water and feed. All animal experiments were approved by the Institute of Zoology Institutional Animal Care and Use Committee.

### Cells lines

HeLa cells, TMCO1 KD HeLa cells and HEK-293 T cells were cultured in Dulbecco’s modified Eagle’s medium (DMEM) with 10% fetal bovine serum (FBS) at 37 °C in a 5% CO_2_ environment. TMCO1 KD HeLa cells were described previously [33].

### UPRE-mCherry reporter construction

UPRE-mCherry reporter has been previously described, which occurred subsequent to endogenous XBP1 splicing [43]. The UPRE promoter region contains 5 UPR elements (UPREs, 5′-TGACGTGG-3′) upstream of the c-fos minimal promoter (−53 to + 45 of the human c-fos promoter) [102]. Finally, mCherry was cloned adjacent to UPRE.

### Lentivirus production

For lentivirus production, HEK-293 T cells were co-transfected with lentivirus vector and packaging plasmids psPAX2 and pMD.2G using PEI. Following 48–72 h of transfection, lentivirus particles were collected for experiments.

### RNA isolation and qRT‒PCR

Total RNA from cells or tissues was extracted by TRIzol Reagent (Invitrogen, 15596026). RNA (1 μg) was reverse-transcribed with GoStript^™^ Reverse Transcription System (Promega, A5001). Quantitative real-time PCR reactions for target genes were performed using Hieff UNICON^®^ Universal Blue qPCR SYBR Green Master Mix (YEASON, 11184ES03). *Gapdh* was used as a normalization control for mRNA. The primer sequences are shown in Reagents and Tools Table.

### Western blotting assay

Cells or tissues were harvested, washed with phosphate-buffered saline (PBS) and lysed on ice with RIPA buffer (150 mM NaCl, 50 mM Tris–HCl, pH 8.0, 1% NP-40, 0.1% sodium dodecyl sulfate (SDS), and 0.5% sodium deoxycholate) containing 50 mM DTT, 1 mM EDTA, 1 mM PMSF, 1 mM NaF and protease inhibitor cocktail (Roche). Then, lysates were centrifuged at 15,000 rpm for 10 min at 4 °C. The total protein was separated by SDS‒PAGE and incubated with primary antibodies for 1 h at room temperature and then at 4 °C overnight. Finally, secondary antibodies conjugated with horseradish peroxidase were incubated for 2 h at room temperature. The bands were visualized by chemiluminescence (Millipore). Density measurements of bands were performed in ImageJ.

### Cycloheximide (CHX) chase assay

Cells were treated with 25 μg/ml CHX and harvested at the indicated time points. Lysates were analyzed by 10% SDS‒PAGE followed by immunoblotting.

### SUnSET assay

SUnSET assay was performed as described previously [42]. Cells were incubated with 10 μg/mL puromycin at 37 °C for 10 min, washed with PBS, and lysed for western blotting with anti-puromycin antibody (Merck, Cat#MABE343).

### Immunoprecipitation

Cells were lysed in NETN buffer (100 mM NaCl, 1 mM EDTA, 20 mM Tris–HCl, pH 8.0, 0.5% NP-40) containing protease inhibitor cocktail (Roche). After incubation for 1 h at 4 °C, the total cell lysates were centrifuged at 4 °C. For pulldown of FLAG-tagged proteins, the cell supernatant was incubated with M2 beads (Sigma‒Aldrich) at 4 °C overnight with a rotating mixer. The next day, the beads were washed with NETN buffer three times. Samples were separated by 10% SDS‒PAGE and analyzed by immunoblotting.

### Protein purification

For IRE1α-LD-FLAG protein purification, a total of three 10-cm dishes of HEK-293 T cells expressing IRE1α-LD-FLAG were lysed in NETN buffer containing protease inhibitor cocktail for 45 min at 4 °C. The total cell lysates were centrifuged at 4 °C, and the cell supernatants were incubated with M2 beads (Sigma‒Aldrich) overnight at 4 °C with a rotating mixer. The beads were washed with NETN buffer, and then the IRE1α-LD-FLAG proteins were eluted with Flag peptide in buffer B (50 mM HEPES, 75 mM NaCl, 10% glycerol, 1 mM TCEP, pH 7.5). The eluents were concentrated and purified with a centrifugal ultrafiltration tube (Millipore, 0.5 ML 30 KD). Finally, the concentration of purified protein was determined by NanoDrop Spectrophotometer (Thermo-Fisher Scientific).

### Pull down assays

For the pulldown assay between GST-BiP-NBD (residues 28–405) [54] and IRE1α-LD-FLAG (residues 1–440) [54], bacterially purified GST-BiP-NBD immobilized on glutathione Sepharose 4B beads was incubated with IRE1α-LD-FLAG protein eluents purified from HEK-293 T cells in buffer B with different concentrations of Ca^2+^ at room temperature for 2 h. After that, the beads were washed with buffer B. And then, IRE1α-LD-FLAG was analyzed by anti-FLAG immunoblotting. Bacterially purified BiP-NBD proteins were visualized by Ponceau S staining.

### Intracellular Ca^2+^ measurement

Intracellular Ca^2+^ measurement was performed as described previously [33]. To measure Ca^2+^ content in the ER, cells were loaded with 4 µM Fura-2 AM (Invitrogen, F1221) and 0.02% pluronic F-127 (Invitrogen, P3000MP) in ECS (140 mM NaCl, 5 mM KCl, 10 mM Glucose, 10 mM HEPES, 2 mM CaCl_2,_ 1 mM MgCl_2,_ pH 7.2–7.4) for 40 min at room temperature in the dark. After three washes with ECS, the cells were incubated for another 10 min in ECS buffer for de-esterification. TG (1 μM) was applied to release Ca^2+^ from the ER in Ca^2+^-free Hank's balanced salt solutions: 140 mM NaCl, 5 mM KCl, 10 mM glucose, 10 mM HEPES, and 1 mM MgCl_2_, pH 7.4. Different Ca^2+^ release inducers were applied in the same manner as TG. Ca^2+^ imaging was taken every 3 s with a Nikon inverted microscope (Eclipse TiE) with a 40 × magnification oil-immersion objective by alternatively excitation at 340 nm and 380 nm. Single-cell Fura-2 images were captured and processed by Metamorph software (version 7.0). The Ca^2+^ level was determined by the 340/380 fluorescence ratio after the Ca^2+^ image background was subtracted. The ER Ca^2+^ content was calculated by the area under the curve induced by the indicated reagents that induce Ca^2+^ release.

### Statistical analysis

Data are shown as the mean ± SEM, and two tailed unpaired Student’s *t* test and one-way ANOVA followed by Tukey’s multiple comparison test were used for statistical significance analysis. All statistical tests were performed by GraphPad Prism 8.

## Supplementary Information

**Additional file 1. **TMCO1 KD leads to the over-activation of IRE1α and regulates RIDD. Related to Fig. 1. (a) IRE1α, p-IRE1α, XBP1s, BiP and CHOP levels were analyzed by immunoblotting in the WT or TMCO1-knockdown cells. GAPDH was used as a loading control. Relative quantification of each protein was shown in the right panel. Bar graphs represent the mean ± SEM from three independent experiments. **P < 0.01, n.s., no significance. (b-d) qRT-PCR analysis of Bip (b), Chop (c) and SPARC (d) mRNA levels in WT or TMCO1 KD cells. Data are shown as mean ± SEM from three independent experiments. n.s., no significance, *** P < 0.001. (e) qRT-PCR analysis of XBP1 mRNA splicing in WT or TMCO1 KD cells treated with 1 μM TG for the indicated times. Bar graphs represent the mean ± SEM from three independent assays. *P < 0.05; **P < 0.01, n.s., no significance. (f) Western blotting analysis of mCherry and XBP1s levels in the HEK-293 T expressing UPRE-mCherry treated with 1 μM TG along with/without 50 μM 4μ8C for 6 h. GAPDH was used as a loading control. Quantification of the relative protein levels are shown in the right panel. Bar graphs represent the mean ± SEM from three independent experiments. **P < 0.01, ***P < 0.001, n.s., no significance. (g) Western blotting analysis of TMCO1 levels after TMCO1 KD in the HEK-293 T. GAPDH was used as a loading control. Relative quantification of each protein was shown in the down panel. Bar graphs represent the mean ± SEM from three independent experiments. *P < 0.05; ***P < 0.001. (h) 1 μM TG-triggered Ca^2+^ transients in WT (purple trace line, n = 300) and TMCO1 KD (red trace line, n = 300) HEK-293 T cells. Each trace line in H is an average of Ca^2+^ responses in each group. Right panel, statistical analysis of the average peak area of TG-triggered Ca^2+^ mobilization curves. ***P < 0.001. (i) IRE1α, XBP1s, TMCO1 levels were analyzed by immunoblotting in the WT or TMCO1 KD HEK-293 T cells. GAPDH was used as a loading control. Relative quantification of each protein was shown in the right panel. Bar graphs represent the mean ± SEM from three independent experiments. **P < 0.01; ***P < 0.001.

**Additional file 2. **Effects of TM and DTT on the ER Ca^2+^ transients. Related to Fig. 2. (a) Assessment of protein synthesis in WT HeLa cells treated with or without BAPTA-AM (100 μM, 2 h) by SUnSET. Data represent as the mean protein intensity normalized to GAPDH ± SEM from 3 independent experiments. ** *P* < 0.01. (b) 3 μg/ml TM or 1 μM TG-triggered Ca^2+^ transients in WT (purple trace line, n = 45) and TMCO1 KD (red trace line, n = 37) cells. Each trace line in B is an average of Ca^2+^ responses in each group. Right panel, statistical analysis of the average peak area of TG-triggered Ca^2+^ mobilization curves. ***P < 0.001. (c) 5 mM DTT or 1 μM TG-triggered Ca^2+^ transients in WT (purple trace line, n = 63) and TMCO1 KD (red trace line, n = 61) cells. Each trace line in C is an average of Ca^2+^ responses in each group. Right panel, statistical analysis of the average peak area of TG-triggered Ca^2+^ mobilization curves. ***P < 0.001.

**Additional file 3. **TMCO1 knockdown leads to the overload of ER Ca^2+^ store. Related to Fig. 3. (a) 100 μM Cch-triggered Ca^2+^ transients in WT (purple trace line, n = 33) and TMCO1 KD (red trace line, n = 25) HeLa cells. Each trace line is an average of Ca^2+^ responses in each group. Right panel, statistical analysis of the average peak area of Cch-triggered Ca^2+^ mobilization curves. Bar graphs represent the mean ± SEM from three independent assays. ***P < 0.001. (b) 1 μM iono-triggered Ca^2+^ transients in WT (purple trace line, n = 42) and TMCO1 KD (red trace line, n = 57) HeLa cells. Each trace line is an average of Ca^2+^ responses in each group. Right panel, statistical analysis of the average peak area of iono-triggered Ca^2+^ mobilization curves. Bar graphs represent the mean ± SEM from three independent assays. ***P < 0.001. (c) 100 μM ATP-triggered Ca^2+^ transients in WT (purple trace line, n = 41), TMCO1 KD (red trace line, n = 44) cells. Each trace line was an average of Ca^2+^ responses in each group. Right panel, statistical analysis of the average peak area of ATP-triggered Ca^2+^ mobilization curves. Bar graphs represent the mean ± SEM from three independent assays. ***P < 0.001. (d) The resting G-CEPIA1er fluorescence ratio signals were detected in WT (n = 454) or TMCO1 KD (n = 540) cells. Bar graphs represent the mean ± SEM from three independent assays. ***P < 0.001.

**Additional file 4. **TMCO1 KD activates IRE1α to prevent cell death. Related to Fig. 6. (a) qRT-PCR analysis of Xbp1s in the liver from 2-month-old WT mice injected intraperitoneally with TM (2 μg/g) for the indicated times. Bar graphs represent the mean ± SEM. Each point represents independent animal. **P < 0.01, ***P < 0.001. (b) Western blotting analysis and quantification of proteins in WT mice injected with TM for the indicated times. GAPDH was used as a loading control. Data represent as the mean protein intensity normalized to GAPDH ± SEM from 3 independent experiments. Each point represents independent animal. *P < 0.05, n.s., no significance. (c) Picrosirius red staining of liver tissues from *Tmco1*^+/+^ *and Tmco1*^−/−^ mice injected with TM (magnification × 200). Scale bar, 100 μm. Three animals per group were analyzed. (d) Western blotting analyses of proteins extracted from liver tissues of *Tmco1*^−/−^ mice and *Tmco1*^+/+^ mice at 8 months of age (three mice per group). GAPDH is used as a loading control. Data represent as the mean protein intensity normalized to GAPDH ± SEM from 3 independent experiments. **P < 0.01.

**Additional file 5: Table S1.** The sources of antibodies used in this study. **Table S2.** The sources of plasmids used in this study. **Table S3.** The sources of primers used in this study. **Table S4.** The sources of chemicals used in this study.

## Data Availability

Data are available upon reasonable request.

## Abbreviations

ER: Endoplasmic reticulum
TMCO1: Transmembrane and coiled-coil domains 1
CLAC: Ca^2+^ load-activated Ca^2+^ channel
IRE1α/ERN1: Inositol-requiring enzyme 1
XBP1: X-box binding protein 1
BiP: Binding immunoglobulin protein
TG: Thapsigargin
ERAD: ER-associated degradation
SERCA: Sarco-/endoplasmic-reticulum Ca^2+^-ATPase
CFTD: Cerebrofaciothoracic dysplasia
miGer: MKate-linker-G-CEPIA1er
qRT-PCR: Quantitative real-time polymerase chain reaction
