# Supplementary figures and images for: ER Ca2+ overload activates the IRE1α signaling and promotes cell survival
Source: Cell Biosci. 2023 Jul 3;13:123. doi: 10.1186/s13578-023-01062-y (PMC10318635; doi:10.1186/s13578-023-01062-y)

Additional file 1

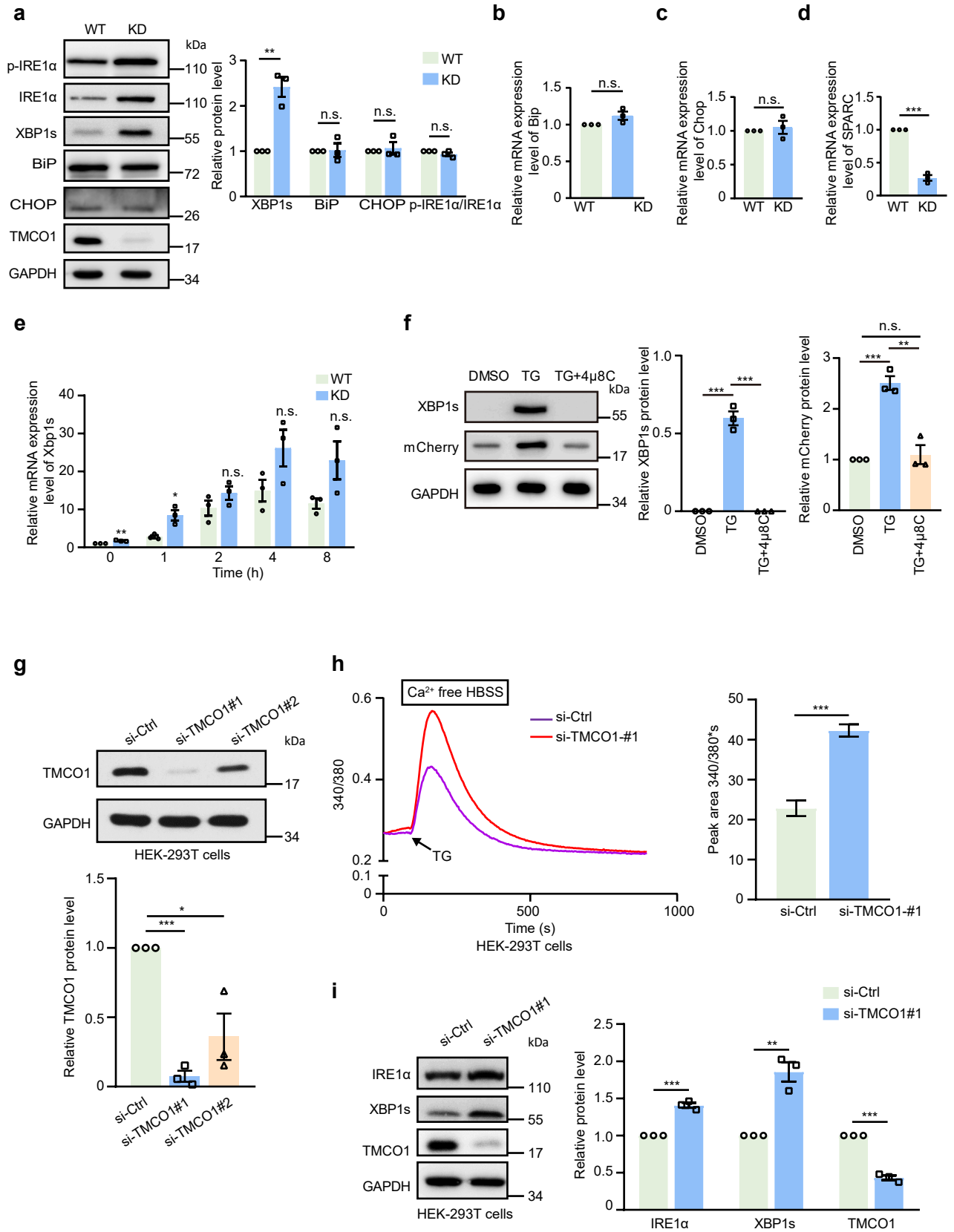

Supplement: Supplementary file 1 — Additional file 1. TMCO1 KD leads to the over-activation of IRE1α and regulates RIDD. Related to Fig. 1. (a) IRE1α, p-IRE1α, XBP1s, BiP and CHOP levels were analyzed by immunoblotting in the WT or TMCO1-knockdown cells. GAPDH was used as a loading control. Relative quantification of each protein was shown in the right panel. Bar graphs represent the mean ± SEM from three independent experiments. **P < 0.01, n.s., no significance. (b-d) qRT-PCR analysis of Bip (b), Chop (c) and SPARC (d) mRNA levels in WT or TMCO1 KD cells. Data are shown as mean ± SEM from three independent experiments. n.s., no significance, *** P < 0.001. (e) qRT-PCR analysis of XBP1 mRNA splicing in WT or TMCO1 KD cells treated with 1 μM TG for the indicated times. Bar graphs represent the mean ± SEM from three independent assays. *P < 0.05; **P < 0.01, n.s., no significance. (f) Western blotting analysis of mCherry and XBP1s levels in the HEK-293 T expressing UPRE-mCherry treated with 1 μM TG along with/without 50 μM 4μ8C for 6 h. GAPDH was used as a loading control. Quantification of the relative protein levels are shown in the right panel. Bar graphs represent the mean ± SEM from three independent experiments. **P < 0.01, ***P < 0.001, n.s., no significance. (g) Western blotting analysis of TMCO1 levels after TMCO1 KD in the HEK-293 T. GAPDH was used as a loading control. Relative quantification of each protein was shown in the down panel. Bar graphs represent the mean ± SEM from three independent experiments. *P < 0.05; ***P < 0.001. (h) 1 μM TG-triggered Ca2+ transients in WT (purple trace line, n = 300) and TMCO1 KD (red trace line, n = 300) HEK-293 T cells. Each trace line in H is an average of Ca2+ responses in each group. Right panel, statistical analysis of the average peak area of TG-triggered Ca2+ mobilization curves. ***P < 0.001. (i) IRE1α, XBP1s, TMCO1 levels were analyzed by immunoblotting in the WT or TMCO1 KD HEK-293 T cells. GAPDH was used as a loading control. Relative quantif [file 13578_2023_1062_MOESM1_ESM.pdf]

## Additional file 2

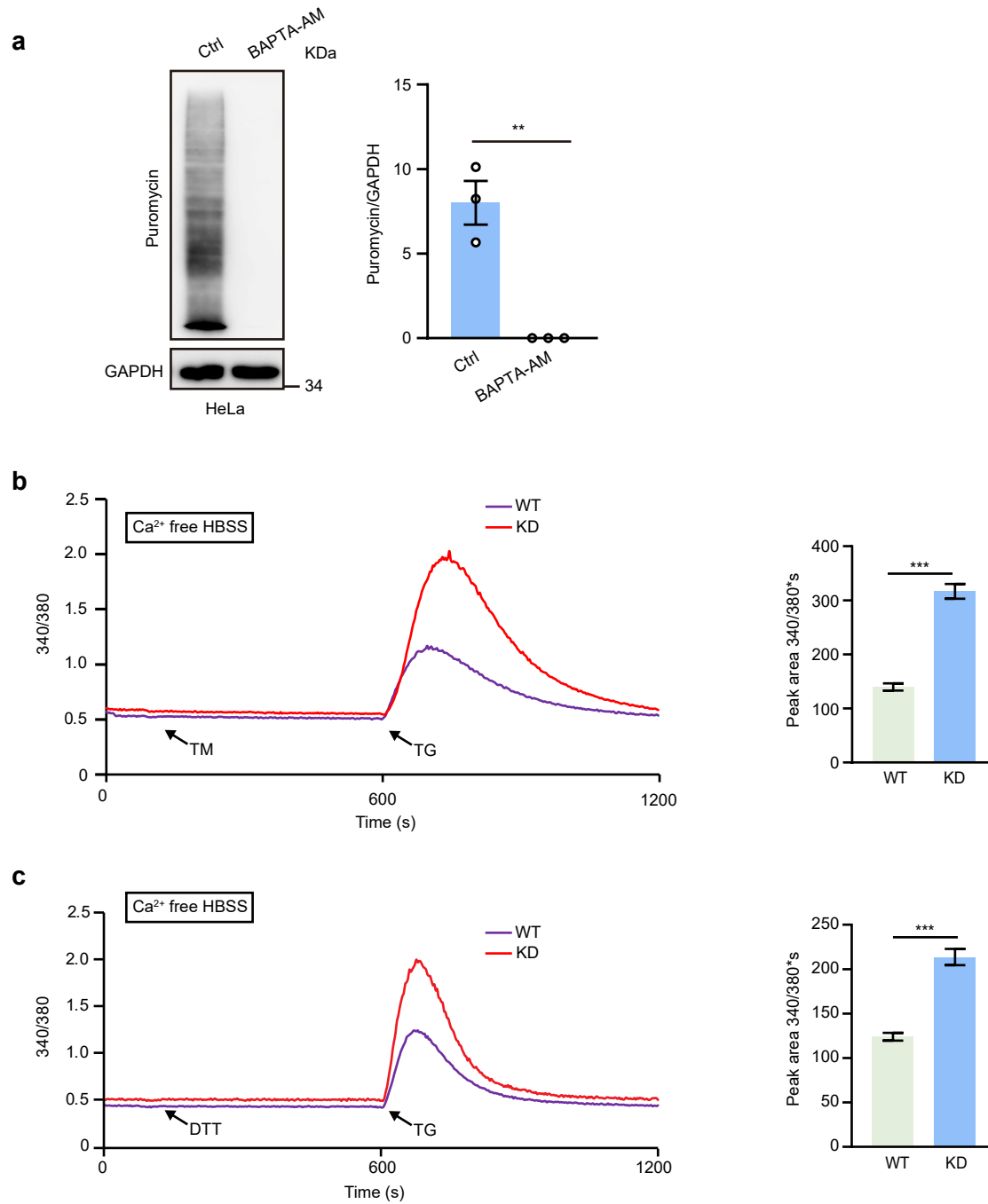

Supplement: Supplementary file 2 — Additional file 2. Effects of TM and DTT on the ER Ca2+ transients. Related to Fig. 2. (a) Assessment of protein synthesis in WT HeLa cells treated with or without BAPTA-AM (100 μM, 2 h) by SUnSET. Data represent as the mean protein intensity normalized to GAPDH ± SEM from 3 independent experiments. ** P < 0.01. (b) 3 μg/ml TM or 1 μM TG-triggered Ca2+ transients in WT (purple trace line, n = 45) and TMCO1 KD (red trace line, n = 37) cells. Each trace line in B is an average of Ca2+ responses in each group. Right panel, statistical analysis of the average peak area of TG-triggered Ca2+ mobilization curves. ***P < 0.001. (c) 5 mM DTT or 1 μM TG-triggered Ca2+ transients in WT (purple trace line, n = 63) and TMCO1 KD (red trace line, n = 61) cells. Each trace line in C is an average of Ca2+ responses in each group. Right panel, statistical analysis of the average peak area of TG-triggered Ca2+ mobilization curves. ***P < 0.001. [file 13578_2023_1062_MOESM2_ESM.pdf]

### Additional file 3

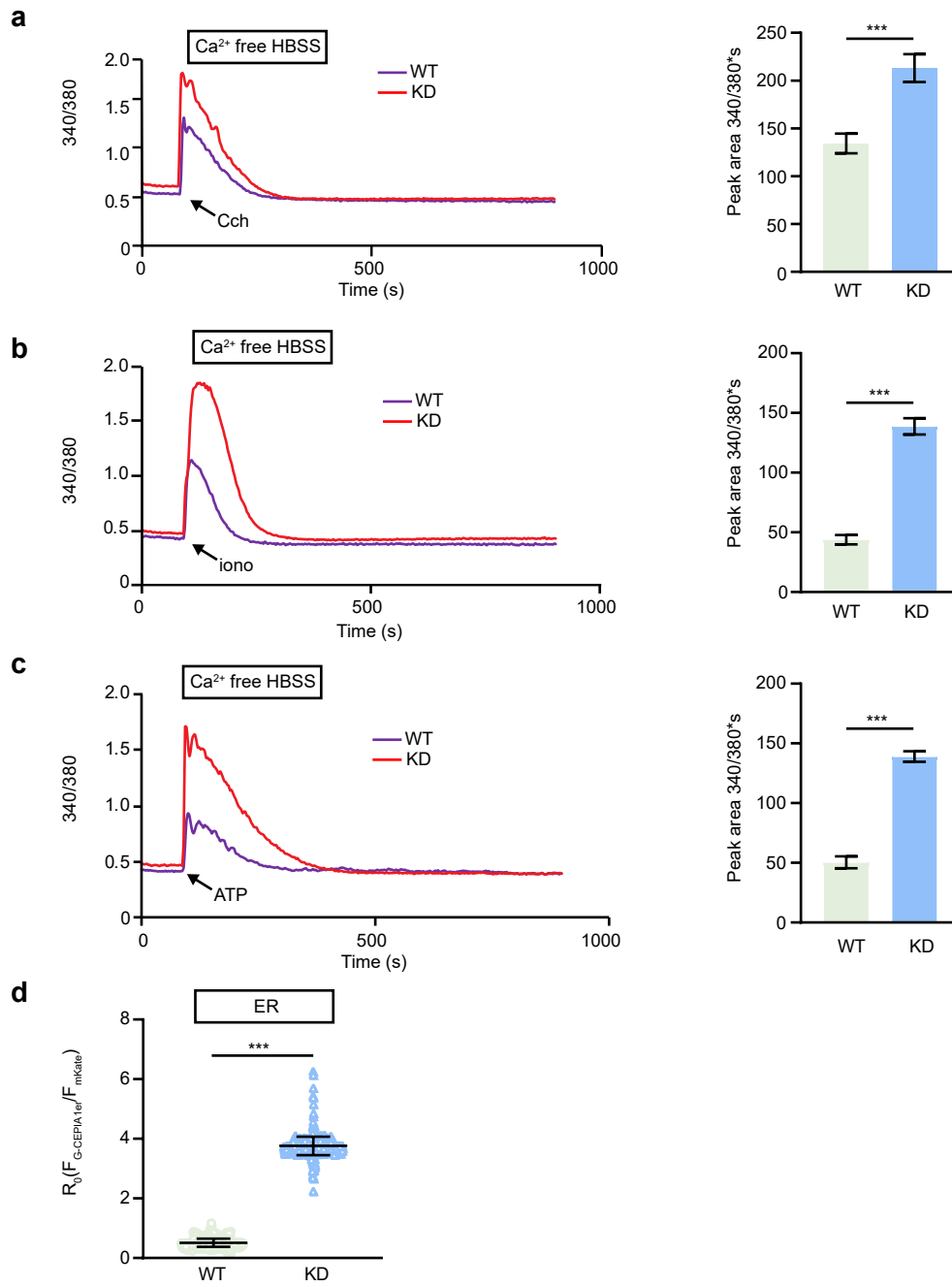

Supplement: Supplementary file 3 — Additional file 3. TMCO1 knockdown leads to the overload of ER Ca2+ store. Related to Fig. 3. (a) 100 μM Cch-triggered Ca2+ transients in WT (purple trace line, n = 33) and TMCO1 KD (red trace line, n = 25) HeLa cells. Each trace line is an average of Ca2+ responses in each group. Right panel, statistical analysis of the average peak area of Cch-triggered Ca2+ mobilization curves. Bar graphs represent the mean ± SEM from three independent assays. ***P < 0.001. (b) 1 μM iono-triggered Ca2+ transients in WT (purple trace line, n = 42) and TMCO1 KD (red trace line, n = 57) HeLa cells. Each trace line is an average of Ca2+ responses in each group. Right panel, statistical analysis of the average peak area of iono-triggered Ca2+ mobilization curves. Bar graphs represent the mean ± SEM from three independent assays. ***P < 0.001. (c) 100 μM ATP-triggered Ca2+ transients in WT (purple trace line, n = 41), TMCO1 KD (red trace line, n = 44) cells. Each trace line was an average of Ca2+ responses in each group. Right panel, statistical analysis of the average peak area of ATP-triggered Ca2+ mobilization curves. Bar graphs represent the mean ± SEM from three independent assays. ***P < 0.001. (d) The resting G-CEPIA1er fluorescence ratio signals were detected in WT (n = 454) or TMCO1 KD (n = 540) cells. Bar graphs represent the mean ± SEM from three independent assays. ***P < 0.001. [file 13578_2023_1062_MOESM3_ESM.pdf]

Additional file 4

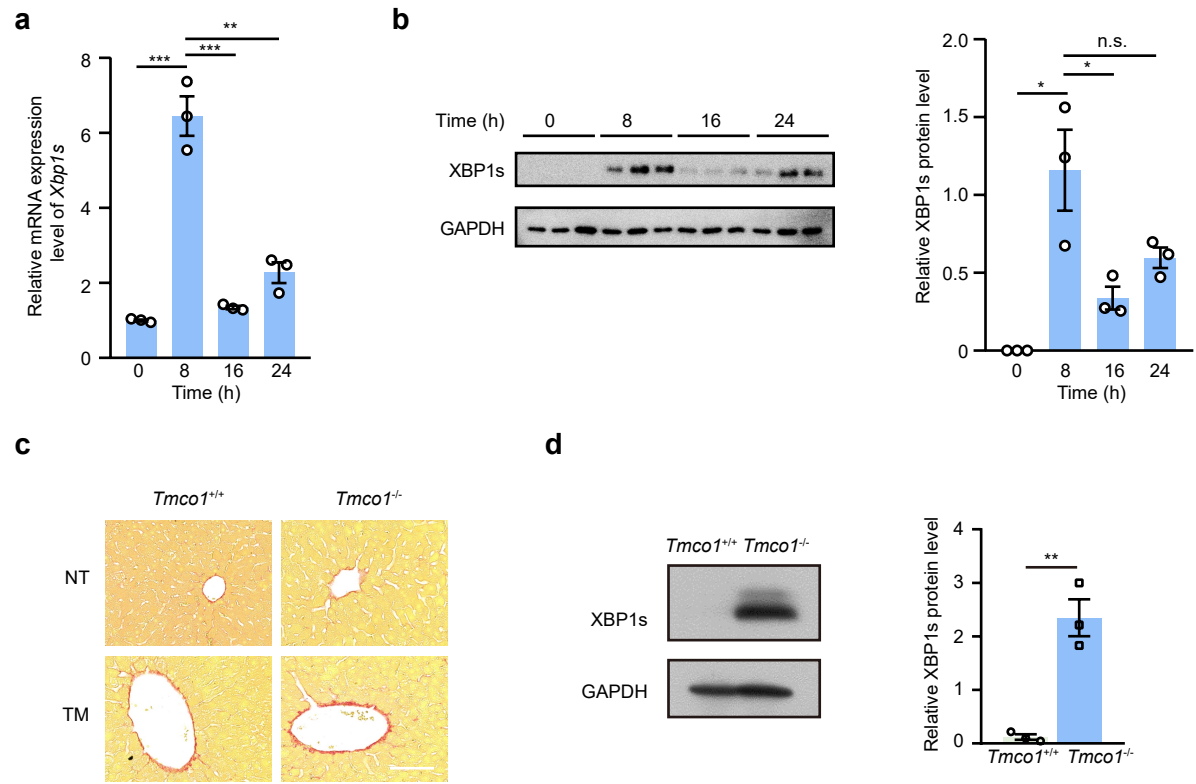

Supplement: Supplementary file 4 — Additional file 4. TMCO1 KD activates IRE1α to prevent cell death. Related to Fig. 6. (a) qRT-PCR analysis of Xbp1s in the liver from 2-month-old WT mice injected intraperitoneally with TM (2 μg/g) for the indicated times. Bar graphs represent the mean ± SEM. Each point represents independent animal. **P < 0.01, ***P < 0.001. (b) Western blotting analysis and quantification of proteins in WT mice injected with TM for the indicated times. GAPDH was used as a loading control. Data represent as the mean protein intensity normalized to GAPDH ± SEM from 3 independent experiments. Each point represents independent animal. *P < 0.05, n.s., no significance. (c) Picrosirius red staining of liver tissues from Tmco1+/+ and Tmco1−/− mice injected with TM (magnification × 200). Scale bar, 100 μm. Three animals per group were analyzed. (d) Western blotting analyses of proteins extracted from liver tissues of Tmco1−/− mice and Tmco1+/+ mice at 8 months of age (three mice per group). GAPDH is used as a loading control. Data represent as the mean protein intensity normalized to GAPDH ± SEM from 3 independent experiments. **P < 0.01. [file 13578_2023_1062_MOESM4_ESM.pdf]
