# Supplementary material for: ER Ca2+ overload activates the IRE1α signaling and promotes cell survival
Source: Cell Biosci. 2023 Jul 3;13:123. doi: 10.1186/s13578-023-01062-y (PMC10318635; doi:10.1186/s13578-023-01062-y)
Supplement: Supplementary file 5 — Additional file 5: Table S1. The sources of antibodies used in this study. Table S2. The sources of plasmids used in this study. Table S3. The sources of primers used in this study. Table S4. The sources of chemicals used in this study. [file 13578_2023_1062_MOESM5_ESM.docx]

**Table S1 The sources of antibodies used in this study**

| Antibodies | Source | Catalog Number |
| --- | --- | --- |
| Rabbit anti-IRE1α | Cell Signaling Technology | Cat #3294 |
| Rabbit anti-XBP1s | Cell Signaling Technology | Cat #40435 |
| Rabbit anti-CHOP | Proteintech | Cat #15204-1-AP |
| Rabbit anti-BiP | Cell Signaling Technology | Cat #3177 |
| Rabbit anti-TMCO1 | Proteintech | Cat #27757-1-AP |
| Rabbit anti-p-IRE1α | CUSABIO | Cat #CSB-RA007795A724phHU |
| Rabbit anti-FLAG | Sigma-Aldrich | Cat #F7425 |
| Rabbit anti-XBP1s | Cell Signaling Technology | Cat #82914 |
| Rabbit anti-PERK | Cell Signaling Technology | Cat #5683 |
| Rabbit anti-ATF6 | Novus | Cat #NBP1-75478 |
| Rabbit anti-eIF2α | Cell Signaling Technology | Cat #5324 |
| Rabbit anti-p-eIF2α | Cell Signaling Technology | Cat #3398 |
| Rabbit anti-ATF4 | Novus | Cat #NBP2-67766 |
| Rabbit anti-calnexin | Cell Signaling Technology | Cat #2679 |
| Mouse anti- GAPDH | Proteintech | Cat #60004-1-Ig |

**Table S2 The sources of plasmids used in this study**

| Recombinant DNA | Source | Catalog Number |
| --- | --- | --- |
| PLVX-TMCO1-IRES-mCherry | This study | N/A |
| PLVX-D140A-IRES-mCherry | This study | N/A |
| PLVX-IRES-mCherry-vector | Addgene | Cat #131824 |
| Tag4A-BiP-FLAG | This study | N/A |
| Tag4A-FLAG-vector | Yong Liu Lab | N/A |
| Tag4A-IRE1α-FLAG | Yong Liu Lab | N/A |
| Tag4A-IRE1α-∆LD-FLAG | This study | N/A |
| UPRE-mCherry | Likun Wang lab | Cat #pBA407 |
| GST-BiP-NBD | This study | N/A |
| Tag4A-IRE1α-LD-FLAG | This study | N/A |
| psPAX2 | This study | N/A |
| pMD.2G | This study | N/A |

**Table S3 The sources of primers used in this study**

| Oligonucleotides and other sequence-based reagents | Source | Catalog Number |
| --- | --- | --- |
| mouse *Xbp1s* (qRT-PCR) | This study | Forward: CTGAGTCCGAATCAGGTGCAG  Reverse: GGATTTCATCCGTACCATTGACA |
| mouse *Chop* (qRT-PCR) | This study | Forward: GGAGCTGGAAGCCTGGTATG  Reverse: TGTGCGTGTGACCTCTGTTG |
| mouse *Bip* (qRT-PCR) | This study | Forward: GTCCAGGCTGGTGTCCTCTC  Reverse: GATTATCGGAAGCCGTGGAG |
| mouse *Bloc1s1* (qRT-PCR) | This study | Forward: AAC ACC AAG CCA AGC AGA ACG A  Reverse: TCACCTCATGGTCCAGCTTTCTCT |
| mouse *Gapdh* (qRT-PCR) | This study | Forward: GGATTTGGCCGTATTGGG  Reverse: GTTGAGGTCAATGAAGGGG |
| Human XBP1s (qRT-PCR) | This study | Forward: TGCTGAGTCCGCAGCAGGTG  Reverse: GCTGGCAGGCTCTGGGGAAG |
| Human XBP1u (qRT-PCR) | This study | Forward: TAAAGTTCTGCTTCTGTCGGG  Reverse: CGGGCTGGCCCCTCTCTGGGC |
| Human BiP (qRT-PCR) | This study | Forward: CATCACGCCGTCCTATGTCG  Reverse: CGTCAAAGACCGTGTTCTCG |
| Human CHOP (qRT-PCR) | This study | Forward: CAAGAGGTCCTGTCTTCAGATGA  Reverse: TCTGTTTCCGTTTCCTGGTTC |
| Human IRE1α (qRT-PCR) | This study | Forward: ACTTTGTCATCGGCCTTTGCAG  Reverse: AGTGAGGCCGCATAGTCAAAGT |
| Human SPARC (qRT-PCR) | This study | Forward: TGAGGTATCTGTGGGAGCTAATC  Reverse: CCTTGCCGTGTTTGCAGTG |
| Human EDEM1 (qRT-PCR) | This study | Forward: CGGACGAGTACGAGAAGCG  Reverse: CGTAGCCAAAGACGAACATGC |
| Human GAPDH (qRT-PCR) | This study | Forward: GGAGCGAGATCCCTCCAAAAT  Reverse: GGCTGTTGTCATACTTCTCATGG |
| siTMCO1#1 | This study | sense（5'-3'）: GCCCUAAUGGGAAUGUUCATT |
| siTMCO1#2 | This study | sense（5'-3'）: UCUCUGUACUAUGUCGAUUTT |
| siCtrl | This study | sense（5'-3'）:  UUCUCCGAACGUGUCACGUTT |

**Table S4 The sources of chemicals used in this study**

| Chemicals, Enzymes and other reagents | Source | Catalog Number |
| --- | --- | --- |
| Polyethylenimine Linear **(**PEI**)** MW40000 | YEASEN | Cat #40816ES02 |
| Thapsigargin | Sigma-Aldrich | Cat #T9033 |
| Tunicamycin | Sigma-Aldrich | Cat #5.04570 |
| KIRA6 | TOPSCIENCE | Cat #TQ0076 |
| 4μ8c | Selleck | Cat #S7272 |
| CDN1136 | Selleck | Cat #S6815 |
| 2-APB | Millipore | Cat #100065 |
| Cycloheximide | Cell Signaling Technology | Cat #2112 |
| Actinomycin D | Selleck | Cat #S8964 |
| BAPTA-AM | Sigma-Aldrich | Cat #A1076 |
| DTT | GPC Bio | Cat #AC334 |
| Annexin V-FITC/PI apoptosis detection kit | Zoman Biotechnology | Cat #ZP327-1 |
